# Acknowledgment to Reviewers of *Pathogens* in 2020

**DOI:** 10.3390/pathogens10020094

**Published:** 2021-01-20

**Authors:** Pathogens Editorial Office

**Affiliations:** MDPI AG, St. Alban-Anlage 66, 4052 Basel, Switzerland

Peer review is the driving force of journal development, and reviewers are gatekeepers who ensure that *Pathogens* maintains its standards for the high quality of its published papers. Thanks to the cooperation of our reviewers, in 2020, the median time to first decision was 14 days and the median time to publication was 2.5 days. The editors would like to express their sincere gratitude to the following reviewers for their precious time and dedication, regardless of whether the papers were finally published:
Abbate, Jessica MariaLi, ChunhaoAbdelwhab, El-Sayed MLi, GuangyuAbernethy, DarrellLi, LinAbeysuriya, RomeshLi, MuzhenAbia, Akebe Luther KingLi, TieshiAbuley, IsaacLi, WenboAbu-Niaaj, LubnaLi, XuguangAcharya, ArpanLiang, JunAckerley, davidLiao, PengAdam, NovobilskýLiao, Ying-YuAdamek, MikolajLiberato, Claudio DeAdamovicz, Jeffrey J.Liechti, George W.Adams, Leslie GarryLightfoot, Jorge D.Adamus-Białek, WiolettaLim, Chun ShenAddie, DianeLim, Siew PhengAdegbola, RaphaelLin, Chao-NanAdhikari, Bal RamLin, Hai-ShuAdil, MohdLin, TaoAdkar-Purushothama, Charith RajLin, Wei-ChenAdney, DanielleLIN, XIONGHAOAgallou, MariaLin, YibinAgarwal, GauravLin, Yusen EasonAgoro, RafiouLindhout, DickAguilera Correa, John JairoLindsay, David S.Ahlenstiel, ChantelleLindsay, DianeAhmad, AsrarLing, JiaxinAhmad, ImtiazLing, Maurice H.T.Ahonen, MerjaLinial, MichalAimanianda, VishukumarLink, AlexanderAiolfi, RobertoLinz, BodoAitken-Palmer, CopperLipke, PeterAjay, Amrendra KLittle, Susan E.Akad, FouadLiu, ChangAkashi, MotohiroLiu, Chao-LinAkbar, SaminaLiu, HaolinAkdesir, EzgiLiu, Hung-JenAkgül, BakiLiu, QiangAkiko, OshiroLiu, QingyunAkinyi, Mercy Y.Liu, WeiAkiyama, HisashiLiu, XiaoyunAlan J, WolfeLiu, YanhongAlanio, AlexandreLiu, ZhuomingAlbano, MarcoLivieratos, Ioannis C.Alberto Díaz-Sánchez, AdrianLleo, MarAlbuquerque, AlexandraLloyd, VettAlcendor, Donald J.Lo, Shih-YenAlderson, MarkŁobacz, AdrianaAlduina, RosaŁobocka, MalgorzataAlexander, NealLojkić, MartinaAlexandersson, ErikLong, S. WesleyAlexandropoulou, IoannaLoose, MariaAl-Ezzi, MinanLopes, Ana Patrícia AntunesAlfson, KendraLópez-López, JoséAli, AkhterLópez-Sanmartín, MonserratAli, Ibne K. M.Lorenzo-Morales, JacobAli, ShawkatLoria, Guido RuggeroAliota, MatthewLosada, AnaAl-Isawi, Rawaa H KLourenço-De-Oliveira, RicardoAllen, DavidLoy, John DustinALLEN, KELLYLozach, Pierre-YvesAllen, Lee-Ann H.Lu, Ming-WeiAlmeida, GabrielLucas, SebastianAl-Saleem, FetwehLuce-Fedrow, AlisonAlsford, SamLück, ChristianAlshabib, EbtihalLudwig-Słomczyńska, Agnieszka H.Altan, EdaLühken, RenkeAltmann, FriedrichLUI, Grace CYAlves, CarlosLukac, DavidAlzan, HebaLukes, JuliusAmarasiri, MohanLundstrom, KennethAmaro, FranciscoLutter, ErikaAmasheh, SalahLütticken, RudolfAmato, FeliceLynn, GeoffreyAmbagala, ArunaMa, ZhengxinAmoutzias, GrigorisMaaß, SandraAn, Dong-JunMabbott, NeilAna, Juan-GarcíaMaccallini, CristinaAnastasiou, Olympia E.Machado, IdalinaAncuceanu, RobertMaciel, MiltonAnděra, LadislavMaciver, SutherlandANDOH, MasakoMackenstedt, UteAndolfi, AnnaMackenzie, CharlesAndonov, AntonMacLean, Oscar AAndrade, Eugénia DeMacRaild, ChristopherAndré, Marcos RogérioMadeira De Carvalho, Luis ManuelAndréani, JulienMadesis, PanagiotisAndreev, Dmitriy NikolaevichMaeda, AkihikoAndrés, María T.Maekawa, ShunAndrews, Simon C.Magan-Fernandez, AntonioAndrophy, ElliotMagyar, DonatAndrukhov, OlehMahony, Timothy JohnAndrzejczuk, SylwiaMahsoub, HassanAngelici, Maria CristinaMaiega, HamidouAngkawidjaja, ClementMaisch, TimAnis, EmanMajer, AnnaAnisimov, Andrey P.Majkowska-Skrobek, GrażynaAnna, MrzljakMajoros, GaborAnstead, Gregory M.Makepeace, BenjaminAnton, HalinaMakita, KoheiAntonelli, AlbertoMaksimov, PavloAntonets, KirillMalbon, AlexandraAntunes, Sandra Isabel Da ConceiçãoMalczewska, BeataApostolakos, IliasMalik, RichardAppell, MichaelMalleret, BenoitAprotosoaie, Ana ClaraMallo, NataliaArabatzis, MichaelMaloney, JennyArango Duque, GuillermoMalwal, Satish R.Aranguren, Luis FernandoMan, AdrianArciello, AngelaMancianti, FrancescaArgaw, TakeleManciulli, TommasoArias, CarolinaManet, EvelyneArien, Kevin K.Manfredi, Maria TeresaArmalyte, JulijaMani, RinoshArndt, Patrick G.Manna, SamAsa’ad, FarahManning, JessicaAsante, EmmanuelManolescu, Loredana Sabina CorneliaAuguste, Albert J.Mansell, PeterAugustine, Swinburne A. J.Maple, PeterAung, Meiji SoeMaranesi, MargheritaAwasthi, MayankaMarc, DanielAwatade, Nikhil TMarc, GabrielAwosile, BabafelaMarceau, MichaelAzam, Aa HaerumanMarchesi, IsabellaAznar, Francisco JavierMarchwińska, KatarzynaAzuma, YasutakaMarcon, AndreaB. Santos, RitaMarenda, MarcBabiuk, ShawnMargaria, PaoloBach, HoracioMaria Irene, BelliniBachmann, NathanMarin Lopez, AlejandroBadea, MihaelaMarín, ClaraBafort, FrançoiseMarini, GiovanniBagiu, Iulia-CristinaMarino, FabioBajer, AnnaMarkovska, RumyanaBajwa, JangiMarkus, HeimesaatBakaloudis, DimitrisMarocco, AdrianoBaker, JuliaMarois-Crehan, CorinneBakre, Abhijeet A.Marotta, FrancescaBakula, ZofiaMarrakchi, HediaBalasch, MonicaMarruchella, GiuseppeBaldin, ClaraMarsilio, FulvioBaldwin, CynthiaMartin, FabiolaBaldwin, ThomasMartin, NeginBaldy-Chudzik, KatarzynaMartin, PatrickBallini, AndreaMartín-Acebes, Miguel A.Bamford, ConnorMartín-Cuervo, MaríaBanadyga, LoganMartínez Beamonte, RobertoBandopadhyay, RinaMartinez, EricBangham, CharlesMartinez, JuanBannantine, John PMartinez, LuisBanović, PavleMartínez-Avilés, MartaBaptista, Rodrigo De PaulaMartínez-Gómez, PedroBaranov, Maksim V.Martínez-Nova, AlfonsoBarba Recreo, MartaMartínez-Turiño, SandraBarbeau, BenoitMartín-Rodríguez, Alberto J.Barber, StuartMartins, IsabelBarbet, AnthonyMarty, GaryBarcena, JuanMaruyama, HaruhikoBardůnek Valigurová, AndreaMarzec-Grządziel, AnnaBar-Haim, ErezMarzinek, JanBarkham, TimothyMasatani, TatsunoriBaron, Julianne L.Mascitti, MarcoBarrera, RobertoMaskow, ThomasBarry, GeraldMaslov, Dmitri A.Barth, DylanMaslow, Joel N.Barth, ThomasMassart, SebastienBartholomeeusen, Dr. KoenMassey, AndrewBartoloni, AlessandroMastino, AntonioBartosik, KatarzynaMasuda, IsaoBasco, LeonardoMatalobos, María ConstenlaBashkin, James K.Matei, IoanaBasler, ChristopherMatilla, MiguelBasmaciyan, LouiseMatsubara, KeiichiBassetto, César CristianoMatsumoto, YasuhikoBastidas, Robert J.Matsuno, KeitaBastos, Reginaldo G.Matsuyama, TomomasaBasu, RupsaMattana, AntonellaBattah, BasemMatthysse, Ann G.Bauer, MartaMattiucci, SimonettaBawage, SwapnilMatttila, TapaniBazzano, MarilenaMaturana, Carola J.Beamer, Gillian LMaurelli, Maria PaolaBeasley, ShannonMauricio, IsabelBeauclair, GuillaumeMaurin, MaxBeauvais, Wendy A.Mavian, CarlaBeccari, GiovanniMavrogianni, Vasia S.Becchimanzi, AndreaMay, JaredBeck, ReljaMay, MeghanBecker, Stefanie ChristineMayer, RupertBedard, EmilieMayr, AstridBeddoe, TravisMaze, Michael J.Behm, Carolyn A.Mazzola, Silvia MichelaBehrendt, PatrickMbonye, UriBehzadi, PayamMcBride, AlisonBeknazarova, MeruyertMcDonnel, Samantha J.Belardinelli, Juan ManuelMcGill, JodiBellini, Natália KarlaMcGregor, Bethany L.Bellini, RoméoMcGregor, Neil R.Bellini, SilviaMcKenzie, DebbieBello-Espinosa, Luis E.McKinstry, Karl KaiBelloso-Uribe, KepaMcLean, AmyBeloukas, ApostolosMcMahon-Pratt, DianeBelser, JessicaMcMillin, MatthewBelsham, GrahamMechaly, ArielBeltrame, AnnaMedina, Gisselle N.Benakanakere, Manjunatha R.Medo, JurajBenazzi, CinziaMehaffy, CarolinaBender, Scott C.Mehedi, MasfiqueBenedetti, FrancescaMehle, NatasaBenfield, David A.Mehlenbacher, ShawnBenitez, LauraMeijer, Harold J GBenmerzouga, ImaanMeiswinkel, RudolphBenzarti, EmnaMejias Luqu, RaquelBerger, PetyaMelegari, GabrieleBermudez, Luiz E.Melendy, ThomasBermudez, MarcelMelik, WessamBerrilli, FedericaMeliopoulos, Victoria ABerteau, Jean-PhilippeMeneses, Patricio I.Bertelloni, FabrizioMenna-Barreto, Rubem Figueiredo SadokBerthold-Pluta, AnnaMenzo, StefanoBertram, MirandaMesalam, AhmedBerzins, AivarsMescolini, GiuliaBesteiro, SébastienMeshram, ChetanBettencourt, PauloMetras, RaphaelleBeugnet, FredericMeulia, TeaBever, CandaceMeurens, FrancoisBhattacharjee, MrinalMeyer Sauteur, Patrick M.Bhattacharyya, TapanMichel, Magdalena M.Bhave, DevayaniMiddelveen, MarianneBialy, DagmaraMiele, Adriana E.Bianchini, RodolfoMiglio, AriannaBianco, Piero AttilioMihai, MaraBielska, EwaMilenkovic, IvanBiernasiuk, AnnaMillán, JavierBilger, AndreaMillemann, YvesBillmyre, BlakeMiller, Haylea C.Bilska-Zając, EwaMinami, MasaakiBinder, UlrikeMinato, Ken-ichiroBiosca, Elena G.Miranda, CarlaBiron, David GeorgesMiriagou, ViviBírošová, LuciaMir-Sanchis, IgnacioBiscarini, FilippoMishra, DushyantBisharat, NaielMitrea, Ioan LiviuBjørgen, HåvardMittapelly, PriyankaBjörkman, AndersMiura, NaokiBlanco, EstherMiyazaki, MotoyasuBlanco-Lobo, PilarMizuguchi, MarikoBlasco, Jose MariaMlaga, Kodjovi D.Blasdell, KimMlocicki, DanielBlazquez, Ana-BelenMoawad, Amira A.Bleilevens, ChristianModhiran, NaphakBobbo, TaniaMoen, Aina E.F.Bocedi, AlessioMoiré, NathalieBochniarz, MariolaMojtahedi, ZahraBock, C. ThomasMojumdar, KamalikaBodily, JasonMoniuszko, AnnaBoerlage, AnnetteMonsarrat, PaulBøgwald, JarlMontagnaro, SerenaBohannon, Julia K.Monteiro, SilviaBohannon, KevinMonteiro, SílviaBohinc, TanjaMontes, NuriaBojić, MirzaMontoliu, IsabelBojinov, BojinMontresor, AntonioBok, EwaMoody, CaryBolick, DavidMoore, Matthew D.BONDOC, IonelMoore, Roger A.Bonetta, SaraMor, SunilBonetta, SilviaMorales, JorgeBongiorno, DafneMora-Montes, HectorBongiorno, GioiaMoraru, Gail MiriamBonnet, Alejandro SuarezMorbidelli, LuciaBorame Lee, DickensMordecai, GideonBoroń-Kaczmarska, AnnaMorel, Chantal M.Borsetti, AlessandraMorello, LauraBosch, MartaMoreno Mesonero, LauraBose, SayantanMoreno, AnaBosse, Jens B.Morgan, Iain M.Bostik, PavelMorganti, GiuliaBotwright, NatashaMori, MarcellaBouchery, TiffanyMori, YasukoBougard, DaisyMorkunas, IwonaBougdour, AlexandreMorley, ValerieBourquain, DanielMorris, FayeBourque, Daniel LeeMorris, JamesBowen, JamesMorris, MeredithBowen, RichardMorse, DanielBracco, EnricoMossel, EricBraczkowski, RyszardMossong, JoëlBradford, BarryMostafa, AhmedBradfute, StevenMotoie, GabrielaBratton, BenjaminMousa, Jarrod J.Braun, LaurenceMoutailler, SaraBrayton, KellyMoutelíková, RomanaBrenišin, MarekMowlaboccus, ShakeelBretagne, StéphaneMu, YunxiangBreyta, RachelMulenga, AlbertBrezgin, SergeyMüller, JoachimBrien, James D.Müller, NorbertBrites, DanielaMuloi, DishonBrito, MiguelMumcuoglu, Kosta Y.Brnić, DraganMunang’andu, Hetron MweembaBrock, DebbieMunck, IsabelBrodosi, LuciaMunday, JohnBroes, AndréMundo, LuciaBronzo, ValerioMuñoz, PilarBrookes, Sharon MMuñoz-Antoli, CarlaBrown, AngelaMuraille, EricBrown, CharlesMurdaca, GiuseppeBrown, Christopher LyonMurooka, ThomasBruce, Timothy J.Murovska, ModraBrukner, IvanMurphy, BrianBrunson, Emily K.Murray, PaulBrunton, LucyMurray, Shannon M.Bruschi, FabrizioMusch, MarkBubici, GiovanniMusić, Martina ŠerugaBukhari, ZiaMusk, BillBukowski, MichalMustafa, GhazalaBulatov, EmilMutharia, Lucy M.Bulavaitė, AistėMuthu Karuppan, Mohan KumarBungau, SimonaMutiga, Samuel K.Burbank, LindseyMuxel, Sandra MarciaBurbank, Lindsey PriceMwangi, WaithakaBurbick, ClaireN’Gouemo, ProsperBurge, ColleenNa, Hee SamBurk, RobertNaber, KurtBurke, Rachel M.Nagasaka, KazunoriBurnett, EleanorNagayasu, EijiButi, MariaNajdenski, HristoButler, FrancisNakahara, TomomiButtimer, ColinNakanishi, NorikoButtò, StefanoNakao, RyoByrne, AndrewNakashiro, Koh-IchiCabañes, JavierNally, JarlathCabezas-Cruz, AlejandroNanduri, RavikanthCacciotto, CarlaNano, AdelaCaeiro, Maria FilomenaNarasimhan, SukanyaCaggiano, GiuseppinaNardini, RobertoCaimano, Melissa J.Narouei-Khandan, Hossein A.Cal, Antonieta DeNarra, Hema PrasadCalcutt, MichaelNarwani, Tarun JaiRajCaldas, Lucio AyresNatale, AldaCaldwell, GaryNauseef, WilliamCalero-Bernal, RafaelNavas, AlfonsoCalistri, AriannaNechwatal, JanCallaghan, DavidNedbalcová, KateřinaCama, Vitaliano A.Neerukonda, SabarinathCambaza, EdgarNegri, DonatellaCambiazo, VerónicaNelli, RahulCamp, Jeremy V.Nelson, CassandraCampbell, DavidNemchinov, LevCampos, Samuel K.Nemeș, Roxana MariaCampuzano, AltheaNéstor Abreu, AcostaCamus-Bouclainville, ChristelleNeuman, BenjaminCandon, SophieNeupane, SaraswotiCaneiras, CátiaNF, KamaruzzamanCantley, Melissa D.Nicholas, RobinCantos-Barreda, AnaNicolas, Francisco E.Canuti, MartaNicoletti, LoredanaCao, DianjunNiculita-Hirzel, HélèneCapillo, GioeleNiczyporyk, Jowita SamantaCarabeo, Rey A.Niedźwiedzka-Rystwej, PaulinaCaragata, EricNikolopoulos, GeorgiosCarazo, AlejandroNisar, MuhammadCárcamo, GerardoNishide, YudaiCardeti, GiusyNishigaki, KazuoCardinale, MassimilianoNishiguchi, MasamichiCardoso, LuísNisnevitch, MarinaCarluccio, Anna VittoriaNjemini, RoseCarmena, DavidNociari, MarceloCarnaccini, SilviaNogales, AitorCarnegie, RyanNogalski, MaciejCaron, RosemaryNogueira, PauloCarrasco, AdrianoNoiri, YuichiroCarretero, Encarnación G.Nordgren, JohanCarrillo, CatherineNorén, TorbjörnCarrillo, Catherine D.Norimine, JunzoCarrión, MarNovelli, GiuseppeCarroll, James A.Nowakowicz-Dębek, BożenaCarter, CraigNowotny, NorbertCartuche, LuisNshimyimana, Jean PierreCarvajal, Thaddeus M.Nuesch, JurgCarvalho, Danilo OliveiraNüesch, JürgCasola, AntonellaNúñez-Delgado, AvelinoCassidy, JosephNunziata, AngelinaCastaldi, SilvanaNuttall, Patricia ACastel, GuillaumeNzelu, ChukwunonsoCastillo-Gonzalez, Claudia MarcelaO’Callaghan, DavidCastro, ErikaO’Connor, DanielCastro, JoanaO’Hanlon, RichardCastro-Escarpulli, GracielaO’Shea, HelenCatania, SalvatoreObar, Joshua J.Cavallero, SerenaOchsenreiter, TorstenCavanagh, Jorunn PaulineOehlers, StefanCaverly, Lindsay JaneOgongo, PaulCaviglia, Gian PaoloOgórek, RafałCebrián, RubénOguma, KeisukeCech, ThomasOh, Sang-KeunCeci, AndreaOh, Seung-YoonCenni, VittoriaOhlsson, Sophie M.Cepeda, JavierOho, TakahikoCerenius, LageOhshima, TakayukiCerri, DomenicoOhta, ShinjiCervero-Aragó, SílviaOka, YujiCesbron, SophieOkazaki, KatsunoriChabé, MagaliOklinski, Michal Krystian EgelundChaber, Anne-LiseOkorski, AdamChajȩcka-Wierzchowska, WioletaØkstad, Ole AndreasChalker, VictoriaOkunade, Adewole L.Chambers, ThomasOlaru, Octavian TudorelChan, Gary C.Olech, MonikaChandrashekar, AbishekOlivier, ChrystelChang, Chia-YiOlsen, Anne BeritChang, Chien-YiOmar, Ahmad A.Chang, Theresa Li-yunOn, StephenChang, Tsung-HsienOnuma, ManabuChang, Wen-WeiOomens, Antonius (Tom)Chang, Yen-ChenOono, RyokoChao, Yu-ChanOppegaard, OddvarChapara, VenkatOpriessnig, TanjaChapman, Nora M.Ortega-Mora, Luis M.Charco, Jorge MorenoOrtega-Villaizan, Maria Del MarChase, ChristopherOrusa, RiccardoChatzipanagiotou, StylianosOszako, TomaszChaudhary, Dhiraj KumarOtelea, DanChaudhuri, RoyOthumpangat, SreekumarChauhan, NikhilOtranto, DomenicoChaurasia, A. K.Ottesen, EricChaves, Luis FernandoOtulak-Kozieł, KatarzynaChavez, FranciscoOuedraogo, Andre LinChavez-Calvillo, GabrielaOzaltin, KadirChen, ChenPace, Douglas A.Chen, Chih-ChiehPacenti, MoniaChen, Chun-ChiPaczkowski, JonChen, Hui-ChenPadhi, SoumeshChen, Hui-WenPadmanabhan, JagannathChen, Li LiPaduch, RomanChen, LibenPadzik, MarcinChen, Wen-HsiangPaeshuyse, JanChen, XianmingPaganelli, RobertoChen, Yi-NingPage, WendyChen, ZhaoPal, ChandanChiang, Yu-ChungPallecchi, LuciaChiaradia, ElisabettaPallesen, JesperChifiriuc, Mariana CarmenPalotas, AndrasChifiruc, Mariana CarmenPaltrinieri, SaverioChisu, ValentinaPaluzzi, Jean-Paul V.Chiu, Cheng-HsunPan, YoulianChluba, JohannaPandit, SantoshChoi, Jong SooPanebianco, AntonioChoi, Kyoung SeongPanfil, AmandaChoi, MyeongjinPanning, MarcusChoi, Won HyungPanwar, PriyankaChoi, Won SukPanzella, LuciaChoi, Yong JunPaolino, GiovanniChoi, Youkyung H.Papadopoulos, Elias G.Chomyszyn-Gajewska, MariaPapagiannitsis, Costas C.Chong, RogerPapathanakos, GeorgiosChoudhuri, SubhadipPape, ThomasChow, Chun YuenPapi, PieroChow, Yen-HungPapp-Wallace, Krisztina M.Chowdhury, Imran HussainParamythiotis, SpirosChowdhury, NityanandaParés-Casanova, Pere M.Chowdhury, Piklu RoyPark, DanielChristodoulides, MyronPark, Hee-SooChristodoulou, Maria-IoannaPark, Sang WookChromy, DavidParker, JosieChu, ChishihParratt, KirstenChu, Chun HungParreira, RicardoChuang, Hung-YiParthasarathy, AnutthamanChuang, Kuo-PinParvez, MohammadChuang, Wen-YuPascucci, IlariaChung, Hee-ChunPasdeloup, DavidChurchward, ColinPassantino, GiuseppeCicco, Emiliano DiPasternak, GerardCichero, ElenaPasternak, Taras P.Cilia, GiovanniPastorino, PaoloCillo, FabrizioPatel, HardikkumarCimica, VelascoPatel, NiketaCissoko, MadyPatil, VeerupaxagoudaCiszewski, Wojciech MichałPatrauchan, Marianna A.Citterio, BarbaraPaul, Narayan ChandraClaesson, RolfPavloudi, ChristinaCłapa, TomaszPawar, KamleshClark, SherriePawlak, AleksandraClement, CristinaPaynter, JanineCobine, Paul A.Pease, Anna S.Cocuzza, ClementinaPechous, RogerCoelho, CamilaPeckham, Gabriel D.Coffeng, Luc E.Pedersen, Finn SkouCohen, DanielPeeples, MarkColebunders, RobertPélandakis, MichelColella, VitoPellicano, RinaldoCollantes-Fernandez, EstherPellon, AizeCollina, SimonaPenades, JoseComas-Garcia, MauricioPeng, ChenComer, Jason E.Peng, CongyueComoy, EmmanuelPeng, DuoConceição, NatérciaPennone, VincenzoČonková, EvaPenrith, Mary-LouiseConn, David BrucePeralta, José MauroConsuegra, JessikaPerego, RobertaConti, PioPereira, Maria A.Convertino, MatteoPerelomov, Leonid V.Conzelmann, Karl-KlausPérez Artés, EncarnaciónCoon, KerriPérez-Cataluña, AlbaCooper, RolandPérez-Martínez, ClaudiaCorbeil, SergePerez-Pascual, DavidCorcoran, Jennifer APérez-Pascual, DavidCork, SusanPérez-Sánchez, RicardoCorneau, AurelienPero, RaffaelaCornelis, PierrePerrucci, StefaniaCorrea, MaríaPeter, Emanuel KarlCorreia Coelho, Ana CláudiaPetitot, Anne-SophieCorrente, MarialauraPhalak, PoonamCorrò, MichelaPhalen, DavidCorzo-León, Dora E.Phan, TungCosoveanu, AndreeaPiana, AndreaCosta Moutinho, FabíolaPiantino Ferreira, Antonio JoséCosta, AdrianaPicado, CésarCosta, MatheusPichon, MaximeCosta-Da-Silva, André LuisPickett, AndyCote, ChristopherPicot, StephaneCoupé, StéphanePietrantonio, Patricia VCowley, JeffPietrella, DonatellaCrawford, LindseyPighetti, Gina M.Creamer, RebeccaPikalo, JuttaCrespo, IrenePiñeiro, LuisCristiano, Maria De LurdesPiñero, José ECrossan, ClairePinheiro, JorgeCuadra, GiancarloPinior, BeateCubero, MeritxellPinto, SandraCupp, Eddie W.Pinto, TatianaCytryńska, MałgorzataPischke, SvenD’Accolti, MariaPise-Masison, Cynthia A.D’Amico, GianlucaPitkanen, TarjaD’Andrea, Marco MariaPiu, SahaD’Apuzzo, FabriziaPizzorno, MarieD’Arcy, PadraigPlain, KarrenD’Costa, VanessaPlano, Laura DeDabert, MiroslawaPleckaityte, MildaDabrowska, JoannaPobiega, KatarzynaDacheux, LaurentPoirel, LaurentDahl, Jan-UlrikPojskić, MirzaDahle, Maria KPolak, Mirosław PawełDai, XinghongPolakowski, Nicholas J.Dalessandri, DomenicoPolewczyk, AnnaDallares, SaraPollastro, StefaniaDaly, Seth M.Pomara, CristoforoDamiaans, BertPomari, ElenaDamtie, DebasuPondeville, EmilieDanis-Wlodarczyk, KatarzynaPonta, LindaDarbro, JonathanPonte, NunoDart, JohnPopescu, SilvanaDas, RabindraPospíšilová, ŠárkaDas, ShubhagataPotokar, MajaDas, SiddharthaPoulet, HervéDas, TheerthankarPoulin, LucieDatta, RupsaPoulle, Marie-LazarineDaugelavicius, RimantasPoulsen, K.P.David B., NeedlePourcel, ChristineDavid, JohnPoveda, CristinaDavidson, IritPozio, EdoardoDavies, ChristopherPrasad, AbhishekDavis, JamesPratelli, AnnamariaDavydenko, KaterynaPrearo, MarinoDawood, Mahmoud A.O.Pretto, TobiaDawson, ChristopherPrevots, RebeccaDe Bellis, LuigiPreziuso, SilviaDe Benedictis, PaolaPrice, PatriciaDe Bonis, SalvatorePrieto, Ana Maria SahagunDe Breyne, SylvainPrigigallo, Maria IsabellaDe Buhr, NicoleProft, ThomasDe Carolis, ElenaPronost, StephaneDe Curtis, FilippoProroga, Yolande Thérèse RoseDe Grazia, SimonaProsperi, AliceDe Gregorio, ElianaProvenzano, DanieleDe Groof, AdProverbio, DanielaDe La Fe, ChristianPszczółkowska, AgnieszkaDe La Fuente, LeonardoPuchkov, EvgenyDe La Rosa-Arana, Jorge LuisPuglia, GiuseppeDe Los Santos, TeresaPugliese, MichelaDe Martino, LuisaPulliainen, ArtoDe Riek, JanPuolakkainen, MirjaDe Santis, PaolaPustylnikov, SergeyDe Souza, David P.Puzon, Geoffrey J.De Swart, RikPyo, Hyun MiDe Vos, Clazien J.Qin, HuaDea-Ayuela, MaríaQiu, YongjinDea-Ayuela, María AuxiliadoraQuackenbush, SandraDeblanc, CélineQuayle, Alison J.Debnath, AnjanQueiroga, CristinaDegola, FrancescaQuilez, JoaquinDeim, ZoltánRabkin, CharlesDel Gallo, MddalenaRaby, EdwardDel Mar Cendra, MariaRaftery, MartinDel Real, GustavoRagan, IzabelaDell’Oste, ValentinaRaheem, BamideleDella-Fazia, Maria AgneseRahimi, FaridDellamaria, DeboraRahman, HadiarDelooz, LaurentRaikhy, GauravDemangeat, GérardRailey, Ashley F.Denagamage, ThomasRajaram, MurugesanDeng, XufangRajasekharan, Satish KumarDepotter, JasperRajeeve, KarthikaDeshmukh, RupeshRakic, JelenaDeshpande, GauraviRakov, A. V.Desnos-Ollivier, MarieRakov, AlexeyDesselberger, UlrichRakus, KrzysztofDesseyn, Jean-LucRamanathan, PalaniappanDevasundaram, SanthiRamani, KritikaDevoy-Keegan, JasonRamírez, Ana SofíaDevriendt, BertRampersad, SephraDeWitte-Orr, StephanieRampias, TheodorosDhakal, SantoshRana, JyotiDhandayuthapani, SubramanianRanawade, AyushDhar, ArunRanf, StefanieDi Bella, SantinaRanjan, PeeyushDi Caro, AntoninoRasheduzzaman, MdDi Micco, PierpaoloRather, IrfanDiack, Abigail B.Raudabaugh, DanielDiane Valeriano, ValerieRautelin, HilpiDiaz-San Segundo, FaynaRaval, YashDidier, AndreaRavera, IvanDietzgen, RalfRazzuoli, ElisabettaDili, AlexandraReddy, Vishwanatha R.A.P.Dill, VeronikaRego, RyanDillon, Jo-Anne R.Reichel, Michael P.Dimitrellou, DimitraReif, Kathryn E.Dimitrov, KirilReimann, Regina R.Ding, SiyuanRein, AlanDini, PouyaReina, RamsésDiniz, InêsReis, AnaDirk, FriedrichReisner, AndreasDishon, ArnonRequena, Jose MDix, RichardRequena, Jose MaríaDixon, BrianRety, StephaneDixon, LindaReuter, TimDjalali Farahani-Kofoet, RoxanaReynard, OlivierDjuardi, YennyRhayat, LamyaDkhar, Hedwin KitdorlangRibaldone, Davide GiuseppeDmitryjuk, MałgorzataRibeiro, Rita Cláudia CardosoDoki, TomoyoshiRibeiro, Teresa GDoligalska, MariaRicci, GiuseppeDolka, IzabellaRicci, Maria LuisaDombrovsky, AvivRice, Christopher A.Domenech, AnaRichner, JustinDomingo, EstebanRichner, Justin M.Domingues, SaraRiddick, Eric WellingtonDomínguez, Yolanda SáenzRiley, SeanDomínguez-Bernal, GustavoRimstad, EspenDonald, Claire L.Rinaldi, LauraDonat, Francisco Javier SilvestreRinder, MonikaDong, ShengzhangRipellino, PaoloDongho, Ghyslaine Bruna DjeunangRiska, PaulDonnelly, Callum GeorgeRitler, DominicDopazo, Carlos P.Ritter, ManuelDordet Frisoni, EmilieRivas, CarmenDoritchamou, JustinRivera, VictorDorny, PierreRizk, Mohamed AbdoDors, ArkadiuszRizzo, CaterinaDosoky, NouraRoberto Da Silva, EdsonDoty, Jeffrey B.Robin, Arif Hasan KhanDou, ZhichengRobinson, James IanDoucoure, SouleymaneRobledo, Sara M.Dowgier, GiuliaRoby, Justin A.Doyle, Stephen R.Rocchigiani, GuidoDracatos, Peter MichaelRodas, Juan DavidDrago, LorenzoRodgers, JohnDrakulic, JassyRodrigues, Célia F.Drogari-Apiranthitou, MariaRodrigues, RogérioDroscha, CaseyRodríguez González, ÁlvaroDu, KeRodriguez Valle, ManuelDuarte, AidaRodriguez, FernandoDuarte, MargaridaRodriguez-Andres, JulioDuarte, NoéliaRodríguez-Díaz, JesúsDubé, MathieuRodríguez-López, PedroDubińska-Magiera, MagdaRodriguez-Perez, A.Dubois, JuliaRodríguez-Rojas, AlexandroDuggan, JackieRoh, ChanghyunDumetz, FranckRohde, John R.Dumic, IgorRojas, LauraDunowska, MagdalenaRojas-Peña, AlvaroDussault, PatrickRojo, Maria AngelesDutartre, HélèneRolbiecki, LeszekDutta, SanjuctaRollin, EmmanuelDuval, RomainRolo, JoanaDuzgunes, NejatRoman, LabudaDvorak, CherylRomanelli, Maria GraziaDwidar, MohammedRomani, AndreaDzięgielewska, MagdalenaRomano, JuliaDzimianski, JohnRomanò, LuisaDzimira, StanisławRomero-López, CarmenEastwood, GillianRömling, UteEasy, RussellRomoli, OttaviaEbani, Valentina VirginiaRonca, ShannonEbihara, HidekiRoncada, PaolaEconomou, VangelisRoncarati, AlessandraEdreira, Martin M.Roperto, FrancoEgawa, NagayasuRoque, André Luiz RodriguesEgberink, HermanRoques, PierreEhrt, SabineRosa, Bruce A.Eibl, Martha M.Rosales, RubenEick, SigrunRosani, UmbertoElaswad, AhmedRosenheim, Jay A.Eldridge, Matthew James GeorgeRosenke, KyleElena, CriscuoloRosenthal, KenElkheir, NatalieRosowski, EmilyElleder, DanielRos-Santaella, José LuisEllis, JohnRossi, OmarElse, Terry AnnRostad, Christina A.Elsworth, BrendanRoszczenko-Jasinska, PaulaEmami, NoushinRothenburg, StefanEmbers, MonicaRotondo, John CahrlesEmecen-Huja, PinarRoubalova, KaterinaEmran, Dr. Talha BinRougeaux, ClemenceEndersby, NancyRoulston, DallasEngvall, Eva OlssonRoura, XavierEnjuanes, LuisRouth, AndrewEremeeva, MarinaRouthu, Nanda KishoreErler, SilvioRouzine, IgorErmolaeva, SvetlanaRoychoudhury, PavitraErtl, ReinhardRóżalska, BarbaraEscario, JoséRóżańska, AnnaEschbaumer, MichaelRubin, JosephEscudero-Pérez, BeatrizRückert, ClaudiaEslamloo, KhalilRuddock, Lloyd W.Esona, Mathew DRuddraraju, Kasi ViswanatharajuEssani, KarimRuiz De Ybáñez, María RocíoEstensoro, ItziarRuizendaal, EsméeEsteves, Ana CristinaRussell, AlistairEtxebeste, OierRusu, Laura CristinaEugenin, EliseoRyan, FrankÉva, MikóRyan, MichaelFabrizi, FabrizioRybczyńska-Tkaczyk, KamilaFagoonee, SharmilaRybka, Jakub DaliborFalkinham III, Joseph O.Rychlik, IvanFalla, MarikaRyckman, BrentFan, Chia-KwungRyter, KendalFan, LushengRzeżutka, ArturFan, Ming Z.Sadigh, YasharFan, WenchunSadykov, Marat R.Fang, RongSaelens, XavierFang, Yufeng ‘Francis’Sáenz-Romo, María GloriaFaris, RobertSaffarian, SaveezFarnos, OmarŠafratová, MarcelaFarone, Mary B.Sahay, BikashFarzan, VahabSahu, PranavFavier, Anne-LaureSainas, StefanoFazal, ZeeshanSaitoh, KenjiFeechan, AngelaSajid, AndaleebFeher, TamasSakkas, HerculesFeiss, Michael G.Sakoda, YoshihiroFelicetti, TommasoSaláková, MartinaFeng, HuapengSalamon, DominikaFeng, NingguoSalas-Coronas, JoaquínFeng, ZongdiSalata, CristianoFeodorova, Valentina ASalawu, EmmanuelFerra, BartłomiejSaldarelli, PasqualeFerrantelli, VincenzoSaleh, MonaFerrara, FrancescaSalgado-Salazar, CatalinaFerrara, PietroSalinas-Ramos, Valeria B.Ferre, IgnacioSalje, JeanneFerreira, Ana CristinaSalman, M. D.Ferreira, FernandoSalvador, FernandoFerreira, Leonardo M.R.Salzano, Anna MariaFerreira, Manuel MarquesSamardžija, MarkoFerreira, SusanaSamba-Louaka, AscelFerrigo, DavideŠamec, DunjaFerrins, LoriSamten, BukaFibriansah, GunturSánchez Crespo, MarianoFiedler, TomasSánchez Del Pino, Manuel M.Figaj, DonataSanchez Vargas, Luis AlbertoFigueras-Alvarez, OscarSanchez-Barrios, AndreaFigura, NataleSandanayaka, Atula S. D.Fijan, SabinaSándor, Attila DFilipello, VirginiaSandoz, KelsiFini, Mehdi ASandro, RolesuFinkel, JonathanSanso, Andrea MarielFinlaison, Deborah S.Sansom, Fiona M.Fiore, NicolaSantini, AntonelloFiorillo, LucaSantos, JeffersonFirth, Clair L.Santos-Gomes, GabrielaFitzpatrick, JulieSantucci, AnnalisaFlanagan, DennisSaracchi, MarcoFlemington, ErikSaracino, IlariaFletcher, Nicola F.Sarbini, ShahrulFodor, JozsefŠarić, TomislavFoissac, XavierSarkar, AbhijitForn-Cuní, GabrielSarkar, DhrubaForrest, CraigSarker, SubirForsythe, StephenSarnataro, DanielaForth, Jan HendrikSase, AjinkyaForzan, MarioSatish, LakkakulaFoster, TimSato, HiroshiFoster, Toshana LauriaSato, Marcello OtakeFouladkhah, AliyarSato, TatsuoFox, JulieSato, TetsuoFox, LarrySatoh, TadashiFoyle, LeoSauka, DiegoFraile, LorenzoSauter-Louis, CarolaFrancia, Maria EugeniaSautto, Giuseppe A.François, SarahSavaglia, JemmaFrandsen, EllenSawa, TeijiFrank-Kamenetskaya, Olga V.Sawicka, AnnaFrant, MaciejSayardoust, SharielFranzo, GiovanniSaz, Sergio VillanuevaFraser, JohannaScagliarini, AlessandraFratini, FilippoScarpa, FabioFreitas-Almeida, Angela CorreaSchares, GereonFreundt, Eric C.Schaub, Günter A.Frey, CarolineScheid, PatrickFrimodt-Møller, NielsScheres, JacquesFrischauf, IreneSchetters, TheoFrossard, Jean-PierreSchildgen, OlivierFry, LindsaySchirmer, BastianFry, William EarlSchlachter, SamanthaFrye, Jonathan G.Schmidt-Posthaus, HeikeFthenakis, GeorgeSchneider, Elena K.Fuchs, MarcSchneider-Schaulies, JuergenFuehrer, Hans-PeterScholte, Florine E.M.Fuentes, Màrius V.Schuurman, Henk-JanFujimuro, MasahiroSchwan, WilliamFujita, AkikazuSchwarz, LukasFUJIWARA, KeiSchweigkofler, WolfgangFukuda, AkiraSchweizer, MatthiasFuller, Kevin K.Schwermer, HeinzpeterFurfaro, Lucy L.Scortichini, MarcoFurneri, Pio MariaScott, JohnFuruse, YukiŠebela, MarekFuruyama, WakakoSecott, TimGabig-Cimínska, MagdalenaSeeth, Martin ThoGábor, FöldváriSeki, FumioGabrić, DraganaSekiguchi, SatoshiGabrieli, PaoloSellars, MelonyGaffuri, AlessandraSelvaraj, ArokiyarajGaglia, Marta MariaSen Santara, SumitGaglione, RosaSenel, MakbuleGajdács, MárióSerban, Daniela ElenaGajdosik, Martina SrajerSerban, GeorgetaGalkina, Svetlana I.Sergeeva, Oksana A.Gallego, MontserratSerra, Claudia R.Gallego-Lopez, GinaSerra, DiegoGallina, LauraSerracca, LauraGálvez, RosaSeruga, PrzemysławGanesan, Sukirth MSeuberlich, TorstenGanges, LlilianneSevvana, MadhumatiGarbuglia, Anna RosaSeyler, Felix HoppeGarcía-Bujalance, SilviaShabardina, VictoriaGarcía-Davis, SaraShafer, William M.García-Díez, JuanShah, JyotsnaGarcía-R, Juan CarlosShah, PriyaGarcía-Suárez, María Del MarSham, Yuk YGardiner, Laura-JayneSharaf, KariemGarofalo, MariangelaShariat, NikkiGarrido-Maestu, AlejandroSharma, Adhikarimayum LakhikumarGarrill, AshleySharma, ArvindGarry, RobertSharma, AtulGatkowska, JustynaSharma, LavGaudieri, SilvanaShaw, AndrewGaudreault, Natasha NShaw, KirstyGautam, UmaShaw, Liam P.Gavaldà, LauraSheffield, Karin LohrmannGazzonis, Alessia LiberaShelite, Thomas RobertGea-Banacloche, Juan CarlosShen, ShichenGeerlof, ArieShen, YangGeha, SamehSheorey, HarshaGekenidis, Maria-TheresiaSherer, NathanGelli, AngieShestopalov, Alexander MGenovese, CarloSHIAO, SHIN-HONGGentile, EleonoraShield, JennyGeorge, SantoshShimoda, HiroshiGerhauser, IngoShimomura, HirofumiGerlich, WolframShin, Hyun-JinGhafar, AbdulShin, Kwang-SooGhassib, IyaShiokawa, MaiGhelardi, EmiliaShirai, JyunsukeGherman, Calin MirceaShishkoff, NinaGhio, Andrew JShoukry, NaglaaGhosh, SouvikShree, SonalGhosh, SumitShrestha, BhushanGiaccone, ValerioShrestha, DeepakGiallourou, NatasaShridhar, SurekhaGianchecchi, ElenaSieg, MichaelGiannakou, IoannisŠilha, DavidGiannoni, EricSilva, GoncaloGibson, WendySilva, Marcelo SousaGieryńska, MałgorzataSilvan, Jose ManuelGilbert, MartinSimo, LadislavGillespie, JosephSimões, João Carlos CaetanoGilpin, BrentSimões, Marta FilipaGimenez-Lirola, Luis G.Simões, NelsonGiorni, PaolaSimonin, YannickGiovanardi, DavideSinclair, JohnGiuffrè, Angelo MariaSingh, Ajay VikramGiuffrè, MauroSingh, KamalendraGiulia, SicilianoSingh, PratibhaGiusti, IlariaSingh, Puspendra P.Glading, Angela J.Singh, ReemaGladue, DouglasSingh, Rupesh KumarGliniak, MaciejSingh, SukhvinderGlobisch, DanielSingh, VineetGnanasundram, Sivakumar VadivelSinha, AmitGnat, SebastianSinigaglia, AlessandroGniadek, AgnieszkaSirobhushanam, SirishaGoarant, CyrilleSivaprakasam, SathishGoepfert, Paul A.Skliros, DimitriosGoldberg, TonySkov, JakobGoldstein, StephenSkowron, KrzysztofGolender, NataliaSkowrońska, KatarzynaGolender, NatalyaSkyberg, Jerod A.Gomez Escalada, MargaritaSlama, PetrGómez López, PedroSluder, Ann E.Gomez-Chiarri, MartaŠmajs, DavidGomez-Lucia, EsperanzaSmeele, Ludwig E.Gómez-Sebastián, SilviaSmirnov, Alexey V.Gondek, MichałSmith, CharlotteGONGAL, GyanendraSmith, DavidGonzales, Jose LSmith, GregoryGonzalez, GaelleSmith, RichardGonzález, María EugeniaSmułek, WojciechGonzalez, Miguel ToroSnyder, LoriGonzalez-Dunia, DanielŠoba, BarbaraGonzález-Fandos, María ElenaSojka, DanielGonzalez-Reiche, Ana SilviaSolís García Del Pozo, JuliánGordon, Catherine A.Solopov, PavelGorrell, RebeccaSoltani, MehdiGorres, Kelly L.Somma, StefaniaGortázar, ChristianSong, Hyun-OkGosiewski, TomaszSong, JeongminGoswami, SandeepSorrentino, RobertoGoto, YasuyukiSorsa, TimoGotoh, KenjiSotillo, JavierGowda, Siddabasave B.Sousa, Sílvia A.Gozalbo Rovira, Roberto VicenteSozhamannan, ShanmugaGrabiec, AleksanderSpada, EvaGradinaru, Andrei CristianSparger, ElizabethGraham, SheilaSpear, RobertGraham-Brown, JohnSpecht, SabineGrandi, NicoleSpencer, Juliet V.Grass, GregorSpencer, Peter S.Grassmann, AndréSpergser, JoachimGraves, StephenSpidlova, PetraGreco, MartaSpiliopoulou, IrisGreen, TimothySpinazzè, AndreaGregić, MajaSpinu, MarinaGrenouillet, FredericSplichal, IgorGreppi, AnnaSplitter, GaryGresnigt, Mark SSprague, WendyGrevelding, Christoph G.Springer, AndreaGrieco, FrancescoSquartini, AndreaGrier, Jennifer T.Squire, Sylvia AfriyieGriffiths, EmmaSreevatsan, SrinandGrilli, GuidoSridhar, SiddharthGrippi, FrancescaSrinivasan, SwethaGrosso, FilipaSrivastava, SaurabhGroveman, BradleySrivastava, VaibhavGrüner, BeateSroka, JacekGryschek, Ronaldo Cesar BorgesStadejek, TomaszGrywalska, EwelinaStafford, Francis WilliamGrzela, KatarzynaStahelin, Robert V.Gubert, CarolinaŠtajner, NatašaGuberti, VittorioStana, AncaGuerlesquin, FrancoiseStanford, KimGuerre, PhilippeStanford, StephanieGugliandolo, EnricoStapleford, KennethGuignard, GaëtanStapleford, Kenneth A.Guillen Fretes, Rosa MariaStasiak-Różańska, LidiaGuillon, ChristopheStatsyuk, Natalia V.Guillou, SandrineSteger, GertrudGuitard, JulietteStein, Derek R.Gunn-Moore, DanielleSteinhagen, DieterGuo, BingSteinman, AmirGupta, KuldeepStelinski, Lukasz L.Gusev, Nikolai B.Stępień, ŁukaszGuy, Paul L.Stępień-Pyśniak, DagmaraGyula, PéterSter, CélineHaataja, SauliSternberg-Lewerin, SusannaHackstadt, TedSTEVANOVIC, JevrosimaHaese, Nicole N.Steven, Blaire T.Hahn, AlexanderStevens, DavidHai, RongSteyn, AngelaHakami, RaminStolk, Wilma A.Halary, FranckStraková, PetraHallen-Adams, Heather E.Straniero, ValentinaHalperin, John JStreit, AdrianHalvorsen, PriyaStreubel, JanaHamacher, JürgStricker, RaphaelHamilton, PatrickStriker, Rob T.Han, HesongStromberg, ZacharyHan, Hui-JuStrungaru, Stefan-AdrianHan, Jee EunStuart, DashperHang, JunStubenrauch, FrankHankel, JuliaStukelj, MarinaHanon, Jean-BaptisteSuarez, CarlosHao, GuixiaSuárez, Cinta PrietoHappel, Christine M.Subash, SarguruHarada, KazukiSubbiah, JeevaHarazny, Joanna M.Subbian, SelvakumarHarkin, Kenneth R.Subedi, DineshHarper, GinaSubudhi, SonuHarrison, Lisa M.Sud’ina, Galina F.Harrod, RobertSugimura, HaruhikoHartmann, AntonSuhrbier, AndreasHarvey, PetaSui, YongjunHasan, MehediSun, FengjieHasan, ShakirSun, HaiyanHasegawa, HideoSun, WeiHashem, YaserSun, XiangjieHasni, IssamSun, XiaolunHassan, Sammer-ulSundberg, Lotta-RiinaHassan, Sherif T. S.Sundheim, LeifHattori, ToshioSungthong, RungrochHatzakis, AngelosSuperti, FabianaHaubek, DorteSupronienė, SkaidrėHauser, W. AllenSur, SubhayanHavis, NeilSuresh, SubbulakshmiHayhurst, AndrewSurleac, MariusHazlett, KarstenSuzuki, TohruHealy, KristenSwartz, Turner H.Hefferon, Kathleen L.Swidergall, MarcHefferon, Kathleen LauraŚwiętoń, EdytaHeider, DominikSyromyatnikov, Mikhail Y.Heinekamp, ThorstenSzekeres, EdinaHeinonen-Tanski, HelviSzewczyk, KatarzynaHeinrich, StefanSzkaradkiewicz, AndrzejHeinzen, RobertSztuba-Solinska, JoannaHelder, JohannesSzumny, AntoniHeller, MartinSzweda, PiotrHemphill, AndrewSzymanski, ChristineHeng, Nicholas C.K.Szymczak, AleksandraHenriques, Ana RitaTabara, MidoriHepojoki, JussiTai, AndrewHeras, Vanessa Las HerasTailly, ThomasHerbelin, PascalineTajery, ShahinHerder, VanessaTakamatsu, DaisukeHeredero-Bermejo, IreneTakano, TomomiHernández De Rojas, AlmaTAKATA, TomoakiHernandez, JesusTakeda, MakotoHernández-Martínez, PatriciaTakizawa, NaokiHess, BeckyTalarmin, AntoineHetman, BenjaminTalavera, MiguelHewitt, JonathanTallmadge, RebeccaHigashi, TomomiTamber, SandeepHikichi, YasufumiTan, Gene S.Hildebrand, JoannaTan, QiHill, FraserTanaka, YoshikazuHill, JanetTang, QiyiHillyer, JulianTardy, FlorenceHirakawa, HidetadaTartaglia, GianlucaHirsch, Alec J.Tartey, SarangHo, HonTashima, ToshihikoHo, Shih-ChingTaylor, GrahamHobbs, Emma CTaylor, MatthewHoelzle, Ludwig E.Teglas, MikeHofstetter, Amelia R.Tejedor, M. TeresaHojo, FuhitoTemesvari, LeslyHolland, MartinTempera, ItaloHomma, TakujiroTempesta, MariaHöner Zu Siederdissen, ChristophTerahara, KazutakaHong, XupengTeramoto, TadahisaHönig, VáclavTeresa Pérez-Gracia, MaríaHonko, AnnaTerzaghi, WilliamHoover, Timothy R.Tesfaye, WubshetHope, JayneTessitori, MatildeHopken, Matthew W.Testa, AntoninoHorhat, Florin GeorgeTetaud, EmmanuelHorner, AndreasThanki, Anisha MahendraHorton, JohnThannesberger, JakobHorvatits, ThomasThekkiniath, JoseHoshina, TakayukiThomas, Donald B.Hotea, IonelaThomas, JohnHouldcroft, CharlotteThompson, AndrewHove, PaidasheThompson, Andrew J.Hrdy, JiriThomsen, Allan RandrupHribar, LawrenceThorenoor, NithyanandaHristov, Peter IvanovTichaczek-Goska, DorotaHsu, Ping ChihTilocca, BrunoHu, JiafenTimchenko, TatianaHu, YanmeiTimofeev, Vladimir I.Huang, Chao-LiTita, OvidiuHuang, Jason C.Tiwari, Rakesh K.Huang, LeiTkadlec, JanHuang, Shiang-FenTkavc, RokHuang, ShuanglongTodt, DanielHubbard, MichelleTohma, KentaroHuber, Charlotte A.Toledo, AlvaroHuber, VictorTomoiaga, Liliana LuciaHuck, OlivierToranzo, Alicia E.Hughes, Holly R.Torras-Ambròs, JoanHulin, Michelle T.Torres Barcelo, ClaraHurst, Brett L.Torres-Sangiao, EvaHurtado, AnaTotaro, MicheleIaniro, GiovanniToth, ZsoltIatta, RobertaTournu, HélèneIborra, SalvadorTrautwein-Schult, AnkeIbrahim, AhmedTrdan, StanislavIchikawa-Seki, MadokaTreder, KrzysztofIde, KazukiTretina, KyleInglis, G. DouglasTrevisani, MarcelloInturri, RosannaTripp, LianneIonescu, Mihaela IleanaTrivedi, PankajIorio, Ronald M.Trivedi, UrvishIovino, FedericoTroyer, RyanIsakova-Sivak, IrinaTrujillo, JessieIshikawa, TomohiroTrus, IvanIsola, GaetanoTrzciński, KrzysztofIturri, JagobaTsagkari, ErifyliIvanov, Yuri D.Tsai, Kun-HsienIwagami, MoritoshiTsai, Yu-HuanIzasa, HisashiTseng, Chun-ChiehIzdebska, Joanna N.Tsiouris, VasiliosIzquierdo-Bueno, InmaculadaTsouh Fokou, Patrick ValereIzumiya, YoshihiroTumanov, Alexei V.Jabbar, AbdulTurnbull, DionneJackson, Mary DarbyTzeng, Shiang-JongJackson, Sarah E.Tzika, EleniJacobsen, MonicaUllah, IrfanJaimes, Javier A.Unchwaniwala, NuruddinJain, PrashantUnterweger, ChristineJain, VaibhavUpadhya, Manoj AtmaramjiJamann, Tiffany M.Upton, ChrisJamil, TariqUrbán, EditJangra, RohitUversky, VladimirJanowicz, AnnaUwiera, RichardJaramillo Ortiz, José ManuelVALCARCEL, FELIXJarcuska, PeterValentová, KateřinaJarocki, VeronicaValiakos, GeorgeJeffers, VickiValiuskaite, AlmaJella, KishoreValle, Manuel RodriguezJelocnik, MartinaVallée, EmilieJenkins, FrankValm, Alex M.Jenson, IanVamanu, EmanuelJenvey, CaitlinVan Bortel, WimJeong, Byung-HoonVan Cutsem, PierreJeong, Rae-DongVan De Vijver, David AMCJerzsele, ÁkosVan Den Ende, Jef J.Jiang, Jhih-HangVan Den Wollenberg, Diana J. M.Jiang, Rong-SanVan Der Hoek, LiaJin, JingVan Der Sluis, Renée M.Jin, LingVan Etten, JamesJindo, KeijiVan Griensven, JohanJiricek, JanVan Haele, MatthiasJobin, Marie-LiseVan Muiswinkel, Willem B.Johne, ReimarVan Rompay, Koen K.A.Johnson, Dylan MVan Tonder, Andries J Van TonderJohnson, NicholasVannini, AndreaJohnson, Reed F.Varallyay, EvaJohnson, TammiVarani, LucaJones, Richard WVarga, CsabaJores, JörgVarloud, MarieJorgačevski, JernejVarsani, ArvindJørgensen, JorunnVasileiou, Natalia G.C.Josenhans, ChristineVaskelyte, Jolanta-JustinaJoshi, SameerVasquez, Francisco Javier HernandezJoshi, Shivali S.Vautier, SimonJuan Carlos, Espinosa MartínVaz, PaolaJubbal, SandeepVázquez-Campos, XabierJung, Eun MiVelavan, Thirumalaisamy P.Jung, Won HeeVelkov, TonyJuno, JenniferVellios, EvangelosJurzik, LarsVeloo, AlidaKabani, MehdiVenancio, ArmandoKabiljo, JulijanVenerando, AndreaKaclikova, EvaVenier, PaolaKaczmarek, BeataVenkat, HeatherKaddes, AmineVenter, HenriettaKahl, Barbara C.Verani, MarcoKahler, CharleneVerbyla, MatthewKajfasz, JessicaVerma, SmritiKalatzis, Panos G.Vesa, Stefan CristianKalmár, ZsuzsaVeszelka, SzilviaKalogianni, DespinaVeyron-Churlet, RomainKalra, Raj SinghVia, Laura E.Kamei, KaekoVialle, AgatheKamiloglu, SenemViboud, GloriaKaminaka, HironoriVidana Mateo, BeatrizKamitani, ShigekiVieira-Pinto, MadalenaKamysz, ElżbietaVigilato, Marco Antonio NatalKanamoto, TaiseiVijay, Ajay KumarKanda, TatsuoVilibić-Čavlek, TatjanaKandel, Prem PrasadVilla, ChiaraKaneko, GenVilla, LucaKang, Hee Y.Villa, Tomas G.Kanginakudru, SriramanaVillagra-Blanco, RodolfoKanike, NeelakantaVillari, SaraKant, SashiVillar-Piqué, AnnaKar, ShantimoyVillarruel-López, AngélicaKaradjian, GregoryVimberg, VladimirKaramon, JacekVincendeau, MichelleKaranis, PanagiotisVincent, DelphineKaraś, MagdalenaVinje, JanKarbowiak, GrzegorzVipond, CarolineKarim, ShahidVirella, DanielKaroor, VijayaVishe, MaheshKarpiński, MirosławVismarra, AliceKarpiński, Tomasz M.Vitale, FabrizioKaruppannan, Anbu KumarVitalii, TimofeevKarus, AvoVizzini, PriyaKarvouniaris, MariosVlasova, Anastasia N.Kashanchi, FatahVodnar, Dan CristianKasprzak, AldonaVogt, MeganKatani, Robabvon Fricken, MichaelKato, HirotomoVoordouw, MaartenKatsafadou, AngelikiVooslo, WilnaKatsifas, Efstathios A.Voss, Oliver H.Kaushik, AshleshaVossen, JackKaushik, NehaVoth, DanielKavaliauskas, PovilasVotypka, JanKawiak, AnnaVrentas, Catherine E.Kayali, GhaziVu, HiepKayumov, AiratVučković, DarinkaKazimirova, MariaVuono, ElizabethKędzierska-Mieszkowska, SabinaWadas, WandaKeerthirathne, ThiliniWadsworth, Crista B.Keevil, Victoria L.Walczak, MarekKehl, AlexanderWałecka-Zacharska, EwaKeller, LanceWales, AndrewKelly, PatrickWalker, DavidKelly-Hope, LouiseWalker, Kathleen RKenna, Dervla T.D.Walker, MarjorieKennedy, RoyWalker, Robert J.Kentaro, YamadaWalker, ValerieKępa, MałgorzataWallace, John R.Kern, PeterWallace, RhiannonKeyel, Peter A.Wang, DanKhaledian, EhdiehWang, Fun-InKhalid, HattafWang, JuexinKhan, KrishnenduWang, Liang-ChunKhatri, VishalWang, LingKhattab, MuhammadWang, PenghaoKhezri, AbdolrahmanWang, Robert Y.-L.Khodadadi, FatemehWang, ShaobinKhokhani, DevanshiWang, Sheng-FanKibenge, FrederickWang, SiyunKiełkiewicz, MałgorzataWang, YingKim, Byung S.Wang, ZhejunKim, Hye KwonWang, ZhengKim, SeonghoWang, ZhijunKim, Tae-SungWasniewski, MarineKim, Yu ChulWąsowski, JacekKim, YunjeongWatier-Grillot, StéphanieKimura, TakashiWatkins, CraigKinkar, LiinaWdowiak, KamilKirchdoerfer, Robert N.Weger-Lucarelli, JamesKirkwood, CarlWei, Kai-CheKiselevskiy, MikhailWein, AlexanderKitazumi, AiWeiss, SabrinaKitchen, PhilipWelch, Stephen RKiu, RaymondWen, Chiu-MingKjelland, VivianWertz, Philip W.Klaumann, FranciniWessely-Szponder, JoannaKlewicka, ElzbietaWherritt, Daniel J.Klingelhutz, Aloysius J.Whiley, HarrietKlobuch, SebastianWhite, ElizabethKlockiewicz, MaciejWhittington, CarlKlöhn, PeterWhittles, Lilith KKlutsch, JenniferWichgers Schreur, Paul J.Knapp, JennyWicker, EmmanuelKo, Kwan SooWideman, JeremyKobayashi, TetsuroWidera, MarekKobiler, OrenWijnveld, MichielKocisova, AlicaWiley, Clayton A.Koene, Miriam GjWilkes, Rebecca P.Kogut, MichaelWilkinson, KatalinKokkinakis, Emmanouil N.Wilks, Colin ReginaldKolarevic, JelenaWille, HolgerKoloniuk, IgorWillems, LucKönig, AlexanderWilliams, Allison H.Könönen, EijaWilliams, DavidKónya, JózsefWilliams, John VKordowska-Wiater, MonikaWilliams, LisaKormas, Konstantinos Ar.Williamson, Peter R.Kormuth, KarenWilson, Van G.Korona-Glowniak, IzabelaWindle, Henry J.Kosakyan, AnushWiszniewska-Łaszczych, AgnieszkaKoscova, JanaWitola, William HaroldKosik-Bogacka, DanutaWójcik, AnnaKostygov, Alexei Yu.Wojtkowska, MałgorzataKotcon, James B.Wojtyczka, RobertKotta-Loizou, IolyWolny-Koładka, KatarzynaKovatcheva-Datchary, PetiaWong, Sarah Sze WahKowal, KrzysztofWork, ThierryKowalczyk, AleksandraWöstemeyer, JohannesKozieł, EdmundWozniak-Knopp, GordanaKozubowski, LukaszWoźniakowski, GrzegorzKraft, Anke Renate MariaWright, Denis J.Krajewska-Wędzina, MonikaWu, Hung-YiKranz, AngelaXaplanteri, PanagiotaKrause, MaikeXi, ZhiyongKrawczynski, KamilXia, YinglinKrešić, NinaXie, HaiboKreth, JensXin, YinKretschmer, MatthiasXiong, WuKrick, StefanieXiong, XiaoliKroeker, AndreaXu, JianKrol, EwelinaXu, YiKróliczewska, BożenaXue, GuangaiKrömker, VolkerXue, JiaKroon, Erna GeessienXynias, Ioannis N.Krstanović, VinkoYadavalli, RaghavendraKrüger, NadineYaeno, TakashiKrutova, MarcelaYan, DayunKubo, YoshinaoYanagawa, AyaKuczowski, MaciejYanagihara, RichardKudłak, BłażejYanagisawa, NaokoKugelman, Jeffrey R.Yang, QianruKuhn, Jens H.Yang, Shih ChunKuhnert, PeterYang, YuhengKujan, OmarYao, ChaoqunKumar Kundu, JibanYaraki, Mohammad TavakkoliKumar, AnandYasuike, MotoshigeKumar, AshokYavvari, PrabhusrinivasKumar, BinodYi, Young-SuKumar, SantoshYin, ChaoranKumar, ShaileshYip, Bon HamKumar, SujeetYoko, MatsuzakiKumaraswamy, ChitralaYokota, Shin-ichiKuo, Ping-ChungYokoyama, HiroshiKupke, AlexandraYong, Tai-SoonKurth, AndreasYoon, Sun-WooKuryk, LukaszYoshinari, NobuoKusstatscher, PeterYou, HongKuzmina, Natalia A.Yu, FengqunKvitko, BrianYu, JingyouKwak, DongmiYu, NingKwiatkowski, PawelYuan, KuoKwieciński, JakubYuzhakov, Anton G.Kwilas, StevenZabel, MarkKwon, Hyuk-JoonZając, ZbigniewKwon, Oh-DeogZakhartchouk, AlexanderKydd, JuliaZaleśny, GrzegorzL’Ollivier, CoralieZapotoczna, MartaLabajo-González, ElenaZaragoza, WilliamLabroussaa, FabienZaric, SvetislavLaddomada, AlbertoZegan-Barańska, MałgorzataLafon, MoniqueZeichner, Steven L.Lai, Yu-HengZelger, BernhardLakatos, LorantZella, DavideLamas, AlexandreZeman, PetrLambert, JohnZeng, WeiguangLandi, LuciaZé-Zé, LíbiaLange, DirkZhang, DuoLantero, EstherZhang, JileiLara Santos, LúcioZhang, KaiLarrous, FlorenceZhang, LeiLasecka-Dykes, LidiaZhang, LiLatinovic, OlgaZhang, ShixiongLaura, RossoZhang, ShuLaurimäe, TeiviZhang, TingLauzi, StefaniaZhang, WeiLawrence, William S.Zhang, XiaotaoLawson, VictoriaZhang, YijunLazarev, VassiliZhang, ZhaoLeaver, David J.Zhao, LinboLeclerc, DenisZhao, XihongLeclercq, AlexandreZhao, YangLecureux, JonathanZheng, XiaorongLedgerwood, Emily DesmetZhong, CuiqingLee, SangmiZhou, BangjunLee, Tzong-ShyuanZhou, ManLegendre, MathieuZhu, Li (Licky)Lehman, Dara A.Zhu, LuchangLeifels, MatsZhu, WenjunLeitão, Jorge H.Zhu, XueyongLelešius, RaimundasZidovec Lepej, SnjezanaLemmermann, NilsZingg, Jean-MarcLemos, Manuel L.Zintl, AnnettaLenhard, Justin R.Zmora, PawelLeroux, Louis-PhilippeŻmudzki, JacekLetek, MichalZnosko, Brent M.Leverett, BetsyZou, WeiLevy, HaimZou, ZhongweiLewis, Leslie C.Zowalaty, Mohamed E. ElLeymarie, OlivierZuellig, RichardLeyson, ChristinaZüst, RolandLhomme, SébastienZygner, WojciechLi, Chunfeng


